# Investigation of blood group genotype prevalence in Korean population using large genomic databases

**DOI:** 10.1038/s41598-023-42473-8

**Published:** 2023-09-15

**Authors:** Cheol O Bae, Soon Sung Kwon, Sinyoung Kim

**Affiliations:** Department of Laboratory Medicine, Yonsei University College of Medicine, 50-1 Yonsei-ro, Seoul, 03722 South Korea

**Keywords:** Genetics research, Population screening, Genetic variation, Genetic variation, Anaemia, Genetic testing

## Abstract

Blood group antigens, which are prominently expressed in red blood cells, are important in transfusion medicine. The advent of high-throughput genome sequencing technology has facilitated the prediction of blood group antigen phenotypes based on genomic data. In this study, we analyzed data from a large Korean population to provide an updated prevalence of blood group antigen phenotypes, including rare ones. A robust dataset comprising 72,291 single nucleotide polymorphism arrays, 5318 whole-exome sequences, and 4793 whole-genome sequences was extracted from the Korean Genome and Epidemiology Study, Genome Aggregation Database, and Korean Variant Archive and then analyzed. The phenotype prevalence of clinically significant blood group antigens, including MNSs, RHCE, Kidd, Duffy, and Diego, was predicted through genotype analysis and corroborated the existing literature. We identified individuals with rare phenotypes, including 369 (0.51%) with Fy(a−b+), 188 (0.26%) with Di(a+b−), and 16 (0.02%) with Jr(a−). Furthermore, we calculated the frequencies of individuals with extremely rare phenotypes, such as p (0.000004%), Kell-null (0.000310%), and Jk(a−b−) (0.000438%), based on allele frequency predictions. These findings offer valuable insights into the distribution of blood group antigens in the Korean population and have significant implications for enhancing the safety and efficiency of blood transfusion.

## Introduction

Blood group antigens serve as surface markers for red blood cells (RBCs) and play a pivotal role in transfusion medicine. These antigens are determined by specific genes that encode for glycoproteins and glycolipids present in the membranes of RBCs. ABO and Rh blood group systems are the most clinically significant, and numerous other blood group systems have varying degrees of clinical importance. In addition to ABO, several other blood group systems, such as Rh, Kidd, Duffy, and Kell, possess clinically significant antigens that can be associated with hemolytic transfusion reactions caused by irregular antibodies to these antigens. Irregular antibodies, also known as alloantibodies, can develop in response to foreign blood group antigens during pregnancy, transfusion, or transplantation. Alloantibodies pose risks for hemolytic transfusion reactions. Thus, when recipients possess alloantibodies against particular blood group antigens, specialized transfusion strategies, such as selecting antigen-negative donors, are employed to minimize the possibility of hemolytic transfusion reactions^1^. For patients with rare RBC phenotypes, finding compatible RBCs for transfusion can be challenging, potentially affecting the quality of transfusion support^2,3^.

To date, the International Society of Blood Transfusion (ISBT) has reported 44 blood group systems with 354 red cell antigens (December 2022)^4^. Traditionally, the presence of certain blood group antigens has been confirmed using immunological methods. Advances in genetic and molecular technologies have revealed that blood group diversity is commonly caused by differences in blood group genes. Recent advances in molecular methods have enabled the precise identification of blood group antigens.

Reliable data regarding the prevalence of blood group antigens within a population are useful for practical applications in transfusion medicine. This information can help healthcare providers optimize blood transfusion compatibility, ensure an adequate supply of antigen-negative blood products, and manage blood inventories more effectively.

In the Korean population, limited data are available regarding the distribution of blood group phenotypes and genotypes, including extended phenotypes and rare blood group types. Previous studies have determined the genotypical blood group composition of the Korean population^5–9^. However, the overall genotypic prevalence of blood group antigens, especially rare ones, is difficult to determine because of limited sample size.

Large population genomic databases have been accumulated by several research groups, and access to these databases makes various population genomic studies available, including blood group genotype investigations. In the present study, we used these large genomic databases to determine the frequencies of the predicted blood group phenotypes in the Korean population. We also investigated rare blood group phenotypes with a high probability of producing antibodies against high-frequency antigens.

## Materials and methods

### Blood group alleles

The variants in blood group genes that determine blood group antigen changes were investigated using ISBT blood group tables as a reference. Lists of ISBT blood group alleles and relevant genetic information were obtained from www.isbtweb.org/isbt-working-parties/rcibgt/blood-group-allele-tables.html (last accessed May 8th, 2023). A total of 46 blood group alleles, including two erythrocyte-specific transcription factors, *GATA1* and *KFL1*, were listed. Among the blood groups, those for which we could predict the blood group phenotypes using genetic data from each database were selected. Consequently, the ABO, MNS, P1PK, RHCE, Lutheran, Kell, Duffy, Kidd, Diego, Yt, Dombrock, Colton, Landsteiner–Wiener, Cromer, Knops, Indian, Ok, RHAG (Rh-associated glycoprotein), JR, LAN, KANNO, and SID blood groups were included in the analysis. For the selected blood groups, we collected variant information related to the phenotype of each blood group from their respective databases. We obtained allele frequency information for each genotype and calculated the corresponding frequencies by predicting phenotypes based on the genotype.

### Phenotype prediction

To predict the phenotype of each blood group antigen, we relied on the nucleotide sequences at specific genomic locations corresponding to these antigens. The genomic positions relevant to the major antigens are as follows: ABO (*ABO*) at positions c.261 and c.796; RHCE (*RHCE*) at positions c.48, c.307, and c.676; Duffy (*ACKR1*) at c.125; Kidd (*SLC14A1*) at c.838; Ss (*GYPB*) at c.143; MN (*GYPA*) at c.59, c.71, and c.72; Diego (*SLC4A1*) at c.2561; Dombrock (*ART4*) at c.793; JR (*ABCG2*) at c.376; KANNO (*PRNP*) at c.655; and Ok (*BSG*) at c.274.

### Phenotype frequency prediction

Based on the allele frequency, the predicted phenotype frequency was calculated using the Hardy–Weinberg equation. For two antithetical alleles, if we presume p as the frequency of allele “A” and q as the frequency of allele “a”, p + q = 1 and the Hardy–Weinberg equation are expressed as p^2^ + 2pq + q^2^ = 1. The frequencies of the homozygous genotype AA, heterozygous genotype Aa, and homozygous genotype aa are represented as p^2^, 2pq, and q^2^, respectively. This equation was used to predict the population frequency of each phenotype.

### The Korean Genome and Epidemiology Study

The Korean Genome and Epidemiology Study (KoGES) is a large cohort study of Koreans. In addition to epidemiological data, genetic data were obtained from various Korean cohorts. Whole-genome sequencing (WGS) and single nucleotide polymorphism (SNP) array data were provided by CODA in the National Biobank of Korea, the Agency for Disease Control and Prevention, Republic of Korea. In total, 72,291 SNP array data points were collected from community-based (5493 individuals), urban-based (58,693 individuals), and rural-based (8105 individuals) cohorts. SNP array data were produced using the Korea Biobank Array, which encompasses over 833,000 markers, including more than 247,000 rare frequency or functional variants derived from the analysis of over 2500 sequencing datasets from Koreans^10^. In addition to SNP array data, 2897 WGS datasets were investigated.

### Genome Aggregation Database

The Genome Aggregation Database (gnomAD) contains a large volume of genetic data, and gnomAD v2.1.1 is composed of 15,708 genomes and 125,748 exomes, including data from 1909 Koreans, 8068 East Asians (excluding Koreans), and 77,165 Europeans^11,12^. The gnomAD data (v2.1.1) for each blood group gene were obtained from https://gnomad.broadinstitute.org/. We analyzed the genome and exome data of Koreans, East Asians, and Europeans.

### Korean Variant Archive for a reference database of genetic variations in the Korean population

The Korean Variant Archive, a reference database of genetic variation in the Korean population (KOVA), is a large Korean control database^13,14^. KOVA database is a collection of WGS and whole-exome sequencing (WES) data generated from multiple projects. Normal tissue samples were obtained from diverse populations, 40.16% of which were from patients with cancer, 28.4% from healthy parents of patients with rare diseases, and 31.44% from healthy volunteers. After filtering inadequate data from the 6654 original sequencing data, 5305 samples (3409 WES and 1896 WGS) were selected and provided publicly. The KOVA data were obtained from https://www.kobic.re.kr/kova/.

### Ethics statement

We used three anonymized databases (KoGES, gnomAD, and KOVA). No direct contact with the research subjects was involved, and no additional information about them were gathered or searched for. The Institutional Review Board for Human Research, Yonsei University, Severance Hospital, Seoul, Korea, approved this study (approval number: 2022-1239-001) and waived the need for informed consent. All methods were performed in accordance with relevant guidelines and regulations.

## Results

### Predicted blood group phenotype frequencies from large population SNP array

A total of 72,291 SNP array data points were analyzed, and the predicted RBC antigen phenotype frequencies of each blood group according to genotype analysis are summarized in Table 1. For the ABO blood group, individuals with A, B, AB and O phenotypes were observed to be 25,044 (34.65%), 19,676 (27.22%), 8008 (11.08%) and 19,554 (27.05%) respectively. These results are consistent with the ABO blood type distribution reported in the 2022 blood services statistics by Korean Red Cross^15^, as well as with previous study^16^. For the MNS blood group, only the S antigen was available for analysis. Among these, 186 individuals (0.26%) had the SS genotype, which was predicted to be negative for the s antigen. In the RHCE blood group, 31,772 individuals (43.95%) were negative for the c antigen, whereas 8345 individuals (11.54%) were negative for the C antigen. The numbers of individuals who were negative for the E and e antigens were 35,569 (49.20%) and 6540 (9.05%), respectively. In the Duffy blood group, only 369 individuals (0.51%) carried the Fy(a−b+) phenotype. In the Kidd antigen group, 16,721 (23.13%) and 19,669 (27.21%) individuals carried the Jk(a+b−) and Jk(a−b+) phenotypes, respectively. In the Diego blood group, most of the population exhibited the Di(a−b+) phenotype (n = 64,768, 89.59%), and only 7523 (10.41%) carried the Di(a) antigen. Meanwhile, only 188 individuals (0.26%) were negative for the Di(b) antigen. Among other high-frequency antigen blood groups, Jr(a−) was observed in only 16 individuals (0.02%), and KANNO1− was found in only 182 individuals (0.25%).

**Table 1:** Frequencies of blood group antigen phenotype predicted based on Korean SNP array data (n = 72,291).

Group	Phenotype	Individuals	Frequency (%)
ABO*	A	25,044	34.65
	B	19,676	27.22
	AB	8008	11.08
	O	19,554	27.05
S	ss	65,364	90.42
	Ss	6741	9.32
	SS	186	0.26
C	cc	8345	11.54
	Cc	32,174	44.51
	CC	31,772	43.95
E	ee	35,569	49.20
	Ee	30,182	41.75
	EE	6540	9.05
Duffy	Fy(a+b−)	62,334	86.23
	Fy(a+b+)	9588	13.26
	Fy(a−b+)	369	0.51
Kidd	Jk(a+b−)	16,721	23.13
	Jk(a+b+)	35,901	49.66
	Jk(a−b+)	19,669	27.21
Diego	Di(a+b−)	188	0.26
	Di(a+b+)	7335	10.15
	Di(a−b+)	64,768	89.59
Dombrock	Do(a+b−)	732	1.01
	Do(a+b+)	13,040	18.04
	Do(a−b+)	58,519	80.95
JR	Jr(a+)	72,275	99.98
	Jr(a−)	16	0.02
KANNO	KANNO1+	72,109	99.75
	KANNO1−	182	0.25

*72,282 individuals analyzed (9 undetermined results).

### Predicted blood group phenotype frequencies from WGS data

WGS enabled the analysis of blood groups that could not be examined using SNP array data, such as the MN, Kell, Ok, and other high-frequency antigen groups (Lutheran, Yt, Colton, Landsteiner–Wiener, Cromer, Knops, and Indian). The results of the 2897 WGS analyses are summarized in Table 2. For the blood groups included in the SNP array data analysis, similar results were observed for each blood group frequency compared with the SNP array data. Among the blood groups that could not be predicted based on the SNP array data, the MM and NN phenotypes of the MN blood group were observed in 717 (24.75%) and 870 (30.03%) individuals, respectively. For the Ok blood group, all study populations were predicted to have the Ok(a+) phenotype. Unlike the SNP array data analysis, no individuals with the Jr(a−) phenotype were observed possibly because of the smaller sample size of the entire study population. For the Lutheran, Kell, Yt, Colton, Landsteiner–Wiener, Cromer, Knops, and Indian blood groups, all study populations were predicted to have Lu(a−b+), K−k+, Yt(a+b−), Co(a+b−), Lw(a+b−), Cr(a+), Kn(a+b−), and In(a−b+), respectively.

**Table 2:** Frequencies of blood group antigen phenotype predicted based on WGS data (n = 2897).

Group	Phenotype	Individuals	Frequency (%)
MN	MM	717	24.75
	MN	1310	45.22
	NN	870	30.03
S	ss	2627	90.68
	Ss	259	8.94
	SS	11	0.38
C	cc	341	11.77
	Cc	1305	45.05
	CC	1251	43.18
E	ee	1434	49.50
	Ee	1199	41.39
	EE	264	9.11
Duffy	Fy(a+b−)	2468	85.19
	Fy(a+b+)	417	14.39
	Fy(a−b+)	12	0.41
Kidd	Jk(a+b−)	650	22.44
	Jk(a+b+)	1439	49.67
	Jk(a−b+)	808	27.89
Diego	Di(a+b−)	10	0.35
	Di(a+b+)	315	10.87
	Di(a−b+)	2572	88.78
Dombrock	Do(a+b−)	31	1.07
	Do(a+b+)	551	19.02
	Do(a−b+)	2315	79.91
Ok	Ok(a+)	2897	100.00
	Ok(a−)	0	0.00
JR	Jr(a+)	2897	100.00
	Jr(a−)	0	0.00
KANNO	KANNO1+	2892	99.83
	KANNO1−	5	0.17

### Predicted extended blood group phenotype frequencies from WGS data

WGS data provide gene sequences for each individual, allowing the prediction of the extended blood group antigen phenotypes of each individual. These data are shown in Table 3. The most common phenotype combination, observed in 137 cases (4.74%), included MN, ss, CcEe, Fy(a+b−), Jk(a+b+), Di(a−b+), Do(a−b+), and KANNO1+ . Individuals with blood group antigen phenotypes exhibiting a frequency of > 1% accounted for approximately half of the total cases (1403/2897, 48.4%). These individuals were predicted to display the s, Fy(a), Di(b), Do(b), and KANNO1+ antigens. Extended blood antigen phenotypes with a frequency of less than 1% are shown in Supplementary Table S1.

**Table 3:** Extended blood group antigen phenotype predicted based on WGS data (n = 2897).

MNS				RHCE				Duffy		Kidd		Diego		Dombrock		KANNO	Individuals	%
M	N	S	s	C	c	e	E	Fya	Fyb	Jka	Jkb	Dia	Dib	Doa	Dob	KANNO1		
+	+	−	+	+	+	+	+	+	−	+	+	−	+	−	+	+	137	4.73
+	+	−	+	+	−	+	−	+	−	+	+	−	+	−	+	+	136	4.69
−	+	−	+	+	−	+	−	+	−	+	+	−	+	−	+	+	106	3.66
−	+	−	+	+	+	+	+	+	−	+	+	−	+	−	+	+	94	3.24
+	+	−	+	+	−	+	−	+	−	−	+	−	+	−	+	+	85	2.93
+	+	−	+	+	+	+	+	+	−	−	+	−	+	−	+	+	83	2.87
+	−	−	+	+	+	+	+	+	−	+	+	−	+	−	+	+	80	2.76
+	−	−	+	+	−	+	−	+	−	+	+	−	+	−	+	+	70	2.42
+	+	−	+	+	+	+	+	+	−	+	−	−	+	−	+	+	61	2.11
+	+	−	+	+	−	+	−	+	−	+	−	−	+	−	+	+	60	2.07
−	+	−	+	+	−	+	−	+	−	+	−	−	+	−	+	+	57	1.97
+	−	−	+	+	−	+	−	+	−	−	+	−	+	−	+	+	52	1.79
−	+	−	+	+	−	+	−	+	−	−	+	−	+	−	+	+	52	1.79
−	+	−	+	+	+	+	+	+	−	−	+	−	+	−	+	+	48	1.66
+	−	−	+	+	−	+	−	+	−	+	−	−	+	−	+	+	44	1.52
+	−	−	+	+	+	+	+	+	−	−	+	−	+	−	+	+	42	1.45
−	+	−	+	+	+	+	+	+	−	+	+	−	+	+	+	+	37	1.28
+	+	−	+	+	+	+	+	+	−	+	+	−	+	+	+	+	33	1.14
−	+	−	+	+	+	+	+	+	−	+	−	−	+	−	+	+	33	1.14
+	+	−	+	−	+	−	+	+	−	+	+	−	+	−	+	+	32	1.10
+	+	−	+	+	−	+	−	+	−	+	+	−	+	+	+	+	31	1.07
+	+	−	+	+	−	+	−	+	+	+	+	−	+	−	+	+	30	1.04

### Genotype-based estimation of rare blood group phenotypes

Investigation of the frequencies of low-frequency antigen alleles from KoGES, gnomAD, and KOVA enabled us to predict the frequencies of low-frequency antigen phenotypes in the Korean population. The predicted phenotype frequencies were calculated using the Hardy–Weinberg equation and are presented in Table 4. For comparison, the allocated antigen phenotype frequencies of East Asian and European populations, as analyzed from gnomAD, are also provided. Detailed number and frequencies of identified alleles for each antigen are shown in Supplementary Table S2. Alleles associated with the Fy(a−b−), Di(a−b−), and Cr(a−) phenotypes were not observed in any of the three databases. Except for the c.274G>A variant in *BSG* (Ok(a−)), c.376C>T variant in *ABCG2* (Jr(a−)), c.655G>A variant in *PRNP* (KANNO1−), and c.1396T>C variant in *B4GALNT2* (Sd(a−)), all other variant alleles were observed at frequencies of < 0.2%. The frequencies of p, Co(b+), In(a+b−), and Rhnull were predicted to be less than 0.000005%.

**Table 4:** Predicted frequencies of rare blood group phenotypes.

Group	Phenotype	Predicted phenotype frequency		
		Korean (%)*	East Asian (%)**	European (%)**
P1PK	p	0.000004	0.000000	0.000004
Kell	Null	0.000310	0.000176	0.000142
Kidd	Jk(a−b−)	0.000438	0.017780	0.521999
Colton	Co(b+)	0.000002	0.000000	0.171543
Indian	In(a+b−)	0.000004	0.000000	0.000000
Ok	Ok(a−)	0.002116	0.000000	0.000000
RHAG	Rhnull	0.000001	0.000000	0.000001
JR	Jr(a−)	0.031014	0.002288	0.000065
LAN	Lan−	0.000123	0.000174	0.001151
KANNO	KANNO1−	0.262555	0.097632	0.000000
SID	Sd(a−)	0.181149	0.071783	5.931266

*Data analyzed from Korean Genome and Epidemiology Study, Genome Aggregation Database (gnomAD), and Korean Variant Archive.

**Data analyzed from gnomAD.

## Discussion

Previous studies have attempted to elucidate the distribution of RBC antigen phenotypes and genotypes in the Korean population. However, conventional serological methods have proven challenging for examining a wide range of RBC antigens because of the considerable cost, time, and effort required to verify an individual’s RBC antigens using antisera for multiple antigens. Similarly, for the genotyping of RBC antigens, most previous studies obtained data by enrolling patients and conducting genetic testing with their blood samples, primarily based on sequencing using PCR methods or commercial allele-specific probes^6–8^. Consequently, large-scale studies are difficult to conduct, and only a limited number of variants have been investigated. Advancements in genome sequencing techniques have facilitated the conduct of WGS and WES, leading to the compilation of several databases. Using these resources, we were able to investigate the distribution of blood group genotypes in Koreans with the largest sample size to date, including the KoGES database with 2897 individual WGS data, 72,291 SNP array data, 1909 individual gnomAD data, and KOVA data. The distribution of Korean blood group phenotypes predicted by the genotypes obtained from the three databases showed relatively good agreement with each other. In addition, it correlated with previous genotype studies (see Supplementary Table S3).

The investigation of the frequency of rare RBC antigens is significant because it allows transfusion centers to be prepared for unusual instances of transfusions. This approach mitigates the risk of antigen sensitization and the acquisition of irregular antibodies. However, the prevalence of rare blood group antigens in the Korean population has yet to be adequately determined. While these data are lacking, there have been several reports of irregular antibodies to high-frequency antigens in Korea, including anti-PP1Pk, anti-Rh17, anti-Ku, anti-Fy(a), anti-Di(b), anti-Ge, anti-Yk(a), anti-Ok(a), anti-JMH, anti-Jr(a), and anti-Sd(a)^17^. This study revealed the presence and number of individuals with the Fy(a−b+), Di(a+b−), Jr(a−), Sd(a−), and KANNO1− phenotypes in the population. In the present study, the Fy(a−b+) phenotype frequency ranged from 0.21 to 0.51%, which was slightly lower than the ranges in previous studies^7,8,18,19^. The Fy(a−b−) phenotype, which is linked to resistance to malaria, was not observed across the three databases investigated in this study. The prevalence of the Di(a+b−) phenotype is consistent with previous studies^7,8,20^. The Jr(a) antigen is a high-frequency antigen. The Jr(a−) phenotype is predominantly reported in Japan, with an estimated prevalence of 0.05% in the Japanese population^21^. Several reports focused on Jr(a−) and anti-Jr(a) in Korea^17,21^. However, to the best of our knowledge, the prevalence of individuals with the Jr(a−) phenotype in the Korean population has not been investigated. In the KoGES SNP array analysis, 16 individuals exhibited the Jr(a−) phenotype, with a prevalence of 0.02%. Similarly, the prediction of Jr(a−) phenotype prevalence, as calculated using the Hardy–Weinberg equation based on allele frequency, was 0.03%. These frequencies within the Korean population are slightly lower but still show a high degree of similarity to the Japanese prevalence data. Antibodies against Duffy, Diego, and JR have previously been associated with hemolytic disease of the fetus and newborn (HDFN) or hemolytic transfusion reaction (HTR)^17^. Although some suspected HTRs have been reportedly caused by an unusually strong Sda antigen (Sd(a++))^1,22^, anti-Sd(a) is generally considered clinically insignificant because of its extreme rarity in causing HTRs^1^. Consequently, prevalence data for Sd(a−) had not been identified in the Korean population prior to this study. Anti-KANNO1 has been associated with pregnancy in Japanese women, but it is not related to HTR or HDFN^23^. The KANNO1− phenotype has been reported in 0.44% of the Japanese population^24^. Frequency data of the KANNO1− antigen among Koreans are currently lacking. In the present study, the prevalence of the KANNO1− phenotype was 0.25% in KoGES SNP array data, 0.17% in KoGES WGS data, and 0.37% in gnomAD data, all of which are slightly less than the Japanese prevalence.

At the allele level, alleles associated with the p, Kell-null, Ok(a−), Jk(a−b−), and Lan− phenotypes were observed. The Kell blood group characterized by high immunogenicity and antibodies that evoke HTR or HDFN. K+ is rare among Korean and East Asian populations^18,25,26^, and no individuals with the K+ antigen were identified in this study. The Kell-null allele, however, was shown to be 0.18% in total, and the predicted phenotype frequency was 0.000310%, indicating that approximately 150 individuals with the Kell-null phenotype are expected in Korea. Anti-Ok(a) is not associated with HTR or HDFN, but it is deemed clinically significant because its reaction with the Ok(a+) antigen decreases the survival of RBCs^27–29^. The allele frequency was predicted to be 0.46%, resulting in a phenotype frequency of 0.002%. Moreover, alleles associated with Co(b+) and Rhnull were identified, and the Colton and RHAG systems were also considered to be clinically significant antigens that may cause HTR or HDFN^30,31^. Although not observed in the homozygous pattern, the frequency of Jk(a−b−) alleles was estimated to be more than 0.2% in our investigation. Kidd antigens cause delayed HTR in addition to typical HTR, and the Kidd-null phenotype is rarely observed in most ethnic groups^32^. To date, no reports of the Jk(a−b−) phenotype in Korea are available. In East Asian countries, the frequency of the Jk (a−b−) phenotype is expected to be 0.002% in Japanese, 0.023% in Taiwanese, and 0.008% in Chinese^32^. According to the Hardy–Weinberg equation, the prevalence of individuals with the Jk(a−b−) phenotype was predicted in the present study to be 0.0004%, which is much lower than that of their East Asian neighbors (0.0178%). The overall frequency of alleles associated with Lan− was > 0.1%. Anti-Lan, which causes mild-to-severe HTR and HDFN^33^, has not been reported in the Korean population. Only a few Japanese reports on the Lan− phenotype have been published in Asian countries^34^. In the present study, the prevalence of individuals with the Lan− phenotype was predicted as 0.0001%.

When determining the pool of potential donors for patients in need of transfusions involving rare blood phenotypes, estimating the frequency of these rare blood antigen phenotypes necessitates consideration of the concurrent expression of ABO and RhD antigens. Thus, it should be noted that the frequencies of rare blood antigens should be interpreted in the context of the frequencies of ABO and RhD phenotypes, as demonstrated in this study and previous studies^35,36^.

This study has some limitations. First, analyses were limited by each data format. KoGES SNP array data were initially generated to investigate specific gene regions associated with diseases or SNPs common to Koreans. Therefore, only these particular genetic regions were included in the KoGES SNP array data, and variants related to blood group antigens outside of these regions could not be analyzed. gnomAD and KOVA data only provide the frequency of specific variants in the population, complicating the analyses of blood group antigen variations that require two or more variants. Second, there may be discrepancies in phenotype prediction. We predicted blood group antigen phenotypes by investigating specific gene regions associated with blood group antigen expression. Although this investigation was based on established references, such as ISBT working parties, the actual phenotype could not be confirmed. This limitation could cause discrepancies and, thus, inaccuracies in the results to some extent.

In this study, we examined the prevalence of blood group antigens in a Korean population by using various genetic databases. To the best of our knowledge, this study is the most comprehensive blood group genotype analysis conducted in the Korean population. Investigating a large sample size allowed us to provide accurate and representative data on genotype prevalence in Koreans. Furthermore, our study extended beyond the frequency of blood group phenotypes and also explored the extended blood group antigen phenotype frequencies through KoGES WGS data analysis. Importantly, our relatively large sample size enabled the identification of rare antigen-associated alleles, which were either difficult to detect or presumed to be nonexistent in Koreans owing to their extreme rarity. The increased sample size also enhanced our understanding of their potential phenotypic existence. The collated allele and phenotype frequencies from each dataset are expected to be particularly valuable for transfusion centers. Based on the findings of this study, we encourage the continued collection of large genome datasets from the general Korean population or blood donors. A more extensive database will facilitate a more accurate determination of blood group antigen prevalence in Koreans and identification of donors with rare phenotypes, thereby contributing to safer blood transfusion practices.

### Supplementary Information

Supplementary Tables.

## Data Availability

All data are available from the corresponding author upon reasonable request.
