# Supplementary material for: Investigation of blood group genotype prevalence in Korean population using large genomic databases
Source: Sci Rep. 2023 Sep 15;13:15326. doi: 10.1038/s41598-023-42473-8 (PMC10504236; doi:10.1038/s41598-023-42473-8)
Supplement: Supplementary file 1 — Supplementary Tables. [file 41598_2023_42473_MOESM1_ESM.pdf]

Supplementary Table S1. Extended blood group antigen phenotype predicted based on WGS data (n = 2,897) (All data included)

MNS				RHCE				Duffy		Kidd		Diego		Dombrock		Kanno		
M	N	S	s	C	c	e	E	Fya	Fyb	Jka	Jkb	Dia	Dib	Doa	Dob	KANNO	Individuals	%
+	+	-	+	+	+	+	+	+	-	+	+	-	+	-	+	+	137	4.74%
+	+	-	+	+	-	+	-	+	-	+	+	-	+	-	+	+	136	4.70%
-	+	-	+	+	-	+	-	+	-	+	+	-	+	-	+	+	106	3.67%
-	+	-	+	+	+	+	+	+	-	+	+	-	+	-	+	+	94	3.25%
+	+	-	+	+	-	+	-	+	-	-	+	-	+	-	+	+	85	2.94%
+	+	-	+	+	+	+	+	+	-	-	+	-	+	-	+	+	83	2.87%
+	-	-	+	+	+	+	+	+	-	+	+	-	+	-	+	+	80	2.77%
+	-	-	+	+	-	+	-	+	-	+	+	-	+	-	+	+	70	2.42%
+	+	-	+	+	+	+	+	+	-	+	-	-	+	-	+	+	61	2.11%
+	+	-	+	+	-	+	-	+	-	+	-	-	+	-	+	+	60	2.08%
-	+	-	+	+	-	+	-	+	-	+	-	-	+	-	+	+	57	1.97%
+	-	-	+	+	-	+	-	+	-	-	+	-	+	-	+	+	52	1.80%
-	+	-	+	+	-	+	-	+	-	-	+	-	+	-	+	+	52	1.80%
-	+	-	+	+	+	+	+	+	-	-	+	-	+	-	+	+	48	1.66%
+	-	-	+	+	-	+	-	+	-	+	-	-	+	-	+	+	44	1.52%
+	-	-	+	+	+	+	+	+	-	-	+	-	+	-	+	+	42	1.45%
-	+	-	+	+	+	+	+	+	-	+	+	-	+	+	+	+	37	1.28%
+	+	-	+	+	+	+	+	+	-	+	+	-	+	+	+	+	33	1.14%
-	+	-	+	+	+	+	+	+	-	+	-	-	+	-	+	+	33	1.14%
+	+	-	+	-	+	-	+	+	-	+	+	-	+	-	+	+	32	1.11%
+	+	-	+	+	-	+	-	+	-	+	+	-	+	+	+	+	31	1.07%
+	+	-	+	+	-	+	-	+	+	+	+	-	+	-	+	+	30	1.04%
+	+	-	+	+	+	+	-	+	-	+	+	-	+	-	+	+	28	0.97%
+	-	-	+	+	+	+	+	+	-	+	-	-	+	-	+	+	27	0.93%
+	+	-	+	+	+	+	+	+	+	+	+	-	+	-	+	+	25	0.86%
+	+	-	+	+	+	+	+	+	-	-	+	-	+	+	+	+	24	0.83%
-	+	-	+	+	-	+	-	+	-	+	+	-	+	+	+	+	22	0.76%
+	-	-	+	-	+	-	+	+	-	+	+	-	+	-	+	+	21	0.73%
+	+	-	+	-	+	-	+	+	-	-	+	-	+	-	+	+	21	0.73%
+	+	-	+	+	-	+	-	+	-	+	+	+	+	-	+	+	21	0.73%
+	+	-	+	+	-	+	-	+	-	-	+	-	+	+	+	+	21	0.73%
-	+	-	+	+	+	+	-	+	-	+	+	-	+	-	+	+	21	0.73%
-	+	-	+	+	-	+	-	+	+	+	+	-	+	-	+	+	20	0.69%
+	-	-	+	+	+	+	+	+	-	+	+	-	+	+	+	+	19	0.66%
+	-	-	+	+	-	+	-	+	-	+	+	-	+	+	+	+	19	0.66%
+	+	-	+	+	-	+	-	+	-	+	-	-	+	+	+	+	19	0.66%
-	+	-	+	+	-	+	-	+	-	+	-	-	+	+	+	+	19	0.66%
-	+	-	+	-	+	-	+	+	-	+	+	-	+	-	+	+	19	0.66%
+	-	+	+	+	+	+	+	+	-	+	+	-	+	-	+	+	18	0.62%
+	+	+	+	+	-	+	-	+	+	+	+	-	+	-	+	+	18	0.62%
+	+	-	+	+	+	+	+	+	-	+	+	+	+	-	+	+	17	0.59%
+	+	-	+	+	-	+	-	+	+	-	+	-	+	-	+	+	16	0.55%
-	+	-	+	+	-	+	-	+	-	-	+	-	+	+	+	+	16	0.55%

-	+	-	+	+	+	+	+	+	+	+	+	-	+	-	+	+	16	0.55%
+	-	-	+	+	+	+	+	+	-	+	+	+	+	-	+	+	15	0.52%
+	+	-	+	+	+	+	+	+	+	-	+	-	+	-	+	+	14	0.48%
-	+	-	+	-	+	+	+	+	-	+	+	-	+	-	+	+	14	0.48%
+	-	+	+	+	-	+	-	+	-	+	+	-	+	-	+	+	13	0.45%
-	+	-	+	+	-	+	-	+	-	+	+	+	+	-	+	+	13	0.45%
+	-	-	+	+	-	+	-	+	+	+	+	-	+	-	+	+	13	0.45%
-	+	-	+	+	+	+	-	+	-	-	+	-	+	-	+	+	13	0.45%
+	-	-	+	+	+	+	-	+	-	+	+	-	+	-	+	+	12	0.42%
+	+	-	+	+	+	+	+	+	-	-	+	+	+	-	+	+	12	0.42%
+	+	-	+	+	-	+	-	+	+	+	-	-	+	-	+	+	12	0.42%
+	+	-	+	+	+	+	+	+	-	+	-	-	+	+	+	+	12	0.42%
+	-	-	+	+	-	+	-	+	-	-	+	-	+	+	+	+	12	0.42%
+	+	-	+	+	+	+	-	+	-	-	+	-	+	-	+	+	12	0.42%
+	+	+	+	+	+	+	+	+	-	+	+	-	+	-	+	+	12	0.42%
+	+	-	+	-	+	+	+	+	-	+	+	-	+	-	+	+	11	0.38%
+	-	-	+	+	+	+	+	+	-	-	+	-	+	+	+	+	11	0.38%
+	+	-	+	+	+	+	-	+	-	+	-	-	+	-	+	+	11	0.38%
+	+	-	+	+	-	+	-	+	-	-	+	+	+	-	+	+	11	0.38%
+	+	-	+	+	-	+	-	+	-	+	-	+	+	-	+	+	11	0.38%
-	+	-	+	+	+	+	+	+	-	+	+	+	+	-	+	+	11	0.38%
-	+	-	+	+	-	+	-	+	+	+	-	-	+	-	+	+	11	0.38%
+	-	-	+	+	-	+	-	+	-	+	-	-	+	+	+	+	10	0.35%
+	+	+	+	+	+	+	+	+	-	-	+	-	+	-	+	+	10	0.35%
-	+	-	+	+	+	+	+	+	-	-	+	-	+	+	+	+	10	0.35%
-	+	-	+	-	+	-	+	+	-	+	-	-	+	-	+	+	10	0.35%
+	+	-	+	+	+	+	+	+	+	+	-	-	+	-	+	+	9	0.31%
+	+	-	+	-	+	-	+	+	-	+	-	-	+	-	+	+	9	0.31%
+	-	-	+	+	+	+	+	+	-	+	-	-	+	+	+	+	9	0.31%
+	+	-	+	+	-	+	-	+	-	+	+	+	+	+	+	+	8	0.28%
+	-	-	+	+	-	+	-	+	+	-	+	-	+	-	+	+	8	0.28%
-	+	-	+	+	+	+	+	+	-	+	-	-	+	+	+	+	8	0.28%
+	+	+	+	+	-	+	-	+	-	+	+	-	+	+	+	+	8	0.28%
-	+	-	+	+	+	+	+	+	+	+	-	-	+	-	+	+	8	0.28%
+	+	-	+	-	+	-	+	+	+	+	+	-	+	-	+	+	8	0.28%
-	+	-	+	+	-	+	-	+	+	-	+	-	+	-	+	+	8	0.28%
+	+	-	+	+	+	+	+	+	-	+	-	+	+	-	+	+	8	0.28%
+	+	+	+	+	-	+	-	+	-	+	-	-	+	-	+	+	8	0.28%
+	-	-	+	+	-	+	-	+	-	+	-	+	+	-	+	+	7	0.24%
-	+	-	+	-	+	-	+	+	-	+	+	+	+	-	+	+	7	0.24%
+	+	+	+	+	-	+	-	+	-	-	+	-	+	-	+	+	7	0.24%
+	-	+	+	+	+	+	+	+	-	-	+	-	+	-	+	+	7	0.24%
-	+	-	+	+	-	+	-	+	-	-	+	+	+	-	+	+	7	0.24%
+	-	-	+	-	+	-	+	+	-	+	-	-	+	-	+	+	7	0.24%
+	-	+	+	+	-	+	-	+	-	-	+	-	+	-	+	+	7	0.24%
-	+	-	+	+	+	+	+	+	+	-	+	-	+	-	+	+	7	0.24%
-	+	-	+	+	+	+	+	+	-	-	+	+	+	-	+	+	7	0.24%
-	+	+	+	+	-	+	-	+	-	+	+	-	+	-	+	+	7	0.24%

+	-	-	+	+	+	+	-	+	-	+	-	-	+	-	+	+	6	0.21%
+	+	-	+	+	-	+	-	+	+	+	-	-	+	+	+	+	6	0.21%
+	+	-	+	-	+	-	+	+	+	+	-	-	+	-	+	+	6	0.21%
+	+	-	+	+	+	+	-	+	-	+	+	-	+	+	+	+	6	0.21%
+	-	-	+	+	-	+	-	+	-	-	+	+	+	-	+	+	6	0.21%
+	+	-	+	-	+	+	+	+	-	+	+	-	+	+	+	+	6	0.21%
+	-	-	+	+	+	+	+	+	-	-	+	+	+	-	+	+	6	0.21%
-	+	+	+	+	+	+	+	+	-	+	+	-	+	-	+	+	6	0.21%
+	-	-	+	+	-	+	-	+	+	+	-	-	+	-	+	+	6	0.21%
-	+	-	+	+	-	+	-	+	-	+	-	+	+	-	+	+	6	0.21%
-	+	-	+	-	+	-	+	+	-	-	+	-	+	-	+	+	6	0.21%
-	+	-	+	-	+	-	+	+	+	+	+	-	+	-	+	+	6	0.21%
-	+	-	+	+	+	+	+	+	-	+	-	+	+	-	+	+	5	0.17%
+	+	-	+	-	+	-	+	+	-	+	-	-	+	+	+	+	5	0.17%
-	+	-	+	+	+	+	-	+	-	+	-	-	+	-	+	+	5	0.17%
+	-	-	+	+	+	+	+	+	+	-	+	-	+	-	+	+	5	0.17%
+	-	-	+	-	+	-	+	+	-	-	+	-	+	-	+	+	5	0.17%
+	-	+	+	+	+	+	+	+	-	+	-	-	+	-	+	+	5	0.17%
+	+	+	+	+	+	+	+	+	+	+	+	-	+	-	+	+	5	0.17%
+	+	-	+	+	-	+	-	+	+	+	+	+	+	-	+	+	5	0.17%
+	-	-	+	+	-	+	-	+	-	+	+	+	+	-	+	+	5	0.17%
+	+	-	+	-	+	-	+	+	-	+	+	-	+	+	+	+	5	0.17%
+	+	-	+	-	+	+	+	+	-	-	+	-	+	-	+	+	5	0.17%
-	+	-	+	-	+	-	+	+	+	-	+	-	+	-	+	+	5	0.17%
-	+	-	+	+	+	+	-	+	+	+	+	-	+	-	+	+	5	0.17%
-	+	+	+	+	-	+	-	+	-	+	-	-	+	-	+	+	5	0.17%
-	+	-	+	-	+	-	+	+	-	+	+	-	+	+	+	+	5	0.17%
+	-	-	+	-	+	-	+	+	-	+	+	+	+	-	+	+	4	0.14%
+	+	-	+	+	+	+	-	+	-	+	-	-	+	+	+	+	4	0.14%
+	+	+	+	-	+	-	+	+	-	-	+	-	+	-	+	+	4	0.14%
+	+	+	+	+	-	+	-	+	-	+	+	+	+	-	+	+	4	0.14%
+	+	-	+	+	-	+	-	+	+	-	+	-	+	+	+	+	4	0.14%
+	+	-	+	+	+	+	+	+	+	-	+	-	+	+	+	+	4	0.14%
+	-	-	+	+	+	+	+	+	+	+	-	-	+	-	+	+	4	0.14%
+	+	-	+	+	-	+	-	+	+	+	+	-	+	+	+	+	4	0.14%
+	+	-	+	-	+	-	+	+	-	-	+	-	+	+	+	+	4	0.14%
+	+	+	+	+	+	+	+	+	-	+	-	-	+	-	+	+	4	0.14%
+	+	+	+	-	+	-	+	+	-	+	+	-	+	-	+	+	4	0.14%
+	-	-	+	+	+	+	-	+	-	-	+	-	+	+	+	+	4	0.14%
+	-	-	+	+	-	+	-	+	+	+	+	-	+	+	+	+	4	0.14%
+	+	-	+	+	+	+	+	+	+	+	-	-	+	+	+	+	4	0.14%
-	+	-	+	+	-	+	-	+	+	+	+	-	+	+	+	+	4	0.14%
-	+	-	+	+	+	+	-	+	+	+	-	-	+	-	+	+	4	0.14%
-	+	-	+	+	+	+	-	+	-	+	+	-	+	+	+	+	4	0.14%
-	+	-	+	+	+	+	+	+	-	+	+	+	+	+	+	+	4	0.14%
+	-	-	+	+	+	+	+	+	+	+	+	+	+	-	+	+	4	0.14%
+	-	-	+	+	+	+	+	+	-	+	+	+	+	+	+	+	4	0.14%
-	+	-	+	-	+	-	+	+	-	-	+	+	+	-	+	+	4	0.14%

-	+	-	+	+	-	+	-	+	+	+	-	-	+	+	+	+	4	0.14%
+	-	+	+	+	-	+	-	+	+	+	-	-	+	-	+	+	3	0.10%
+	-	-	+	+	+	+	+	+	+	-	+	-	+	+	+	+	3	0.10%
+	+	-	+	-	+	-	+	+	+	+	+	-	+	+	+	+	3	0.10%
-	+	-	+	+	+	+	+	+	-	-	+	+	+	+	+	+	3	0.10%
+	-	-	+	-	+	-	+	+	-	-	+	-	+	+	+	+	3	0.10%
+	+	-	+	-	+	-	+	+	-	+	+	+	+	-	+	+	3	0.10%
-	+	-	+	-	+	+	+	+	-	+	-	-	+	-	+	+	3	0.10%
+	-	+	+	-	+	-	+	+	-	+	+	-	+	+	+	+	3	0.10%
-	+	+	+	+	+	+	+	+	-	+	-	-	+	-	+	+	3	0.10%
+	+	-	+	+	+	+	-	+	-	+	-	+	+	-	+	+	3	0.10%
+	-	-	+	+	+	+	-	+	-	+	+	-	+	+	+	+	3	0.10%
+	-	-	+	+	+	+	+	+	+	+	+	-	+	+	+	+	3	0.10%
+	+	+	+	+	+	+	+	+	-	+	+	-	+	+	+	+	3	0.10%
+	-	-	+	+	+	+	-	+	-	-	+	-	+	-	+	+	3	0.10%
+	-	-	+	-	+	+	+	+	-	+	+	-	+	-	+	+	3	0.10%
+	+	-	+	+	+	+	+	+	+	+	+	+	+	-	+	+	3	0.10%
+	-	-	+	-	+	-	+	+	-	+	+	-	+	+	+	+	3	0.10%
+	-	-	+	-	+	-	+	+	-	+	-	-	+	+	+	+	3	0.10%
+	+	+	+	+	-	+	-	+	-	-	+	+	+	-	+	+	3	0.10%
+	+	-	+	+	+	+	-	+	-	-	+	-	+	+	+	+	3	0.10%
+	+	-	+	+	+	+	+	+	-	+	-	-	+	+	-	+	3	0.10%
+	-	+	+	-	+	-	+	+	-	-	+	-	+	-	+	+	3	0.10%
+	+	-	+	-	+	+	+	+	-	+	-	-	+	-	+	+	3	0.10%
-	+	+	+	+	+	+	+	+	-	-	+	-	+	-	+	+	3	0.10%
+	-	+	+	+	-	+	-	+	-	+	+	+	+	-	+	+	2	0.07%
+	+	+	+	+	+	+	+	+	+	+	-	-	+	-	+	+	2	0.07%
+	-	+	+	+	-	+	-	+	-	-	+	+	+	-	+	+	2	0.07%
+	-	+	+	+	+	+	+	+	+	-	+	-	+	-	+	+	2	0.07%
+	-	-	+	-	+	+	+	+	-	-	+	-	+	-	+	+	2	0.07%
+	+	-	+	+	+	+	-	+	+	+	+	-	+	+	+	+	2	0.07%
-	+	-	+	+	-	+	-	+	-	-	+	-	+	+	-	+	2	0.07%
+	-	+	+	+	-	+	-	+	-	+	+	-	+	+	+	+	2	0.07%
+	-	-	+	-	+	-	+	+	+	+	+	-	+	-	+	+	2	0.07%
-	+	-	+	+	+	+	-	+	-	+	-	+	+	-	+	+	2	0.07%
+	-	-	+	-	+	+	+	+	-	+	+	-	+	+	+	+	2	0.07%
+	+	+	+	-	+	+	+	+	-	+	+	-	+	-	+	+	2	0.07%
+	-	+	+	-	+	-	+	+	-	+	-	-	+	-	+	+	2	0.07%
+	-	-	+	-	+	-	+	+	-	+	+	-	+	+	-	+	2	0.07%
+	-	+	+	-	+	-	+	+	-	+	+	-	+	-	+	+	2	0.07%
+	-	-	+	-	+	-	+	+	-	+	-	+	+	-	+	+	2	0.07%
+	+	-	+	+	+	+	+	+	-	+	+	+	+	+	+	+	2	0.07%
-	+	-	+	+	-	+	-	+	+	+	+	+	+	-	+	+	2	0.07%
+	+	+	+	+	+	+	-	+	+	+	+	-	+	-	+	+	2	0.07%
+	-	+	+	+	-	+	-	+	-	-	+	-	+	+	+	+	2	0.07%
+	+	-	+	+	-	+	-	+	-	-	+	+	+	+	+	+	2	0.07%
+	+	+	+	+	-	+	-	+	-	+	-	+	+	-	+	+	2	0.07%
+	+	-	+	+	+	+	+	+	+	-	+	+	+	-	+	+	2	0.07%

+	+	-	+	+	-	+	-	+	+	+	+	+	+	+	+	+	2	0.07%
+	+	-	+	+	-	+	-	+	-	+	+	-	+	+	-	+	2	0.07%
+	+	-	+	-	+	-	+	+	+	-	+	-	+	-	+	+	2	0.07%
+	+	-	+	-	+	+	+	+	-	+	+	+	+	-	+	+	2	0.07%
+	+	-	+	+	+	+	+	-	+	-	+	-	+	-	+	+	2	0.07%
+	-	+	-	+	+	+	+	+	-	-	+	-	+	-	+	+	2	0.07%
-	+	-	+	+	+	+	+	+	+	+	+	-	+	+	+	+	2	0.07%
+	-	+	+	+	-	+	-	+	-	+	-	-	+	-	+	+	2	0.07%
+	+	+	+	+	-	+	-	+	-	-	+	-	+	+	+	+	2	0.07%
+	+	+	+	+	-	+	-	+	+	-	+	-	+	-	+	+	2	0.07%
+	+	-	+	+	-	+	-	+	+	+	-	+	+	-	+	+	2	0.07%
-	+	+	+	+	-	+	-	+	-	+	+	-	+	+	+	+	2	0.07%
+	+	-	+	+	+	+	+	+	-	+	+	-	+	+	-	+	2	0.07%
+	-	-	+	+	+	+	+	+	-	+	-	+	+	-	+	+	2	0.07%
+	-	-	+	+	+	+	+	+	+	+	-	-	+	+	+	+	2	0.07%
+	+	+	+	-	+	+	+	+	-	-	+	-	+	-	+	+	2	0.07%
-	+	-	+	-	+	+	+	+	+	+	+	-	+	-	+	+	2	0.07%
+	-	-	+	+	-	+	-	+	+	+	-	-	+	+	+	+	2	0.07%
+	-	-	+	+	-	+	-	+	+	-	+	-	+	+	+	+	2	0.07%
+	-	+	+	+	+	+	+	+	-	+	+	-	+	+	+	+	2	0.07%
+	-	+	+	+	+	+	+	+	-	-	+	-	+	+	+	+	2	0.07%
-	+	-	+	+	+	+	+	+	+	+	+	+	+	+	+	+	2	0.07%
+	-	-	+	-	+	-	+	+	-	-	+	+	+	-	+	+	2	0.07%
-	+	+	+	-	+	-	+	+	-	+	+	-	+	-	+	+	2	0.07%
-	+	-	+	-	+	+	+	+	-	+	+	-	+	+	+	+	2	0.07%
+	-	-	+	+	-	+	-	+	-	+	-	+	+	+	+	+	2	0.07%
-	+	-	+	-	+	-	+	+	-	-	+	-	+	+	+	+	2	0.07%
-	+	+	+	-	+	+	+	+	-	+	+	-	+	-	+	+	2	0.07%
+	+	-	+	+	+	+	-	+	+	+	-	-	+	-	+	+	2	0.07%
-	+	-	+	+	+	+	-	+	-	+	-	-	+	+	+	+	2	0.07%
-	+	-	+	+	+	+	+	+	+	-	+	-	+	+	+	+	2	0.07%
-	+	-	+	-	+	+	+	+	-	-	+	-	+	-	+	+	2	0.07%
+	-	-	+	+	-	+	+	+	-	-	+	-	+	-	+	+	2	0.07%
-	+	-	+	+	+	+	-	+	+	+	+	+	+	-	+	+	1	0.03%
+	-	+	+	+	+	+	-	+	-	-	+	-	+	+	+	+	1	0.03%
+	+	+	+	+	+	+	+	+	-	-	+	-	+	+	+	+	1	0.03%
+	+	-	+	+	+	+	-	+	+	-	+	-	+	-	+	+	1	0.03%
+	-	-	+	+	-	+	-	+	+	+	-	+	+	+	+	+	1	0.03%
+	+	-	+	-	+	+	-	+	-	+	-	-	+	-	+	+	1	0.03%
+	+	+	+	+	-	+	-	+	-	+	-	-	+	+	+	+	1	0.03%
+	+	+	+	-	+	-	+	+	+	+	+	-	+	-	+	+	1	0.03%
+	-	-	+	+	+	+	-	+	-	+	-	+	+	-	+	+	1	0.03%
+	+	-	+	+	+	+	-	+	-	+	+	-	+	+	-	+	1	0.03%
+	-	-	+	-	+	-	+	+	-	+	-	+	-	-	+	+	1	0.03%
+	-	+	+	+	+	+	-	+	-	+	+	+	+	-	+	+	1	0.03%
-	+	-	+	+	-	+	-	+	-	+	+	+	+	+	-	+	1	0.03%
-	+	+	+	-	+	+	+	+	-	-	+	-	+	-	+	+	1	0.03%
+	+	-	+	+	+	+	-	+	-	-	+	+	+	-	+	+	1	0.03%

+	-	-	+	+	+	+	+	+	+	-	+	+	+	+	+	+	1	0.03%
+	-	-	+	+	-	+	-	+	+	-	+	+	+	-	+	+	1	0.03%
+	-	-	+	+	-	+	-	-	+	+	+	-	+	-	+	+	1	0.03%
+	+	-	+	-	+	+	-	+	-	+	+	-	+	-	+	+	1	0.03%
+	+	-	+	-	+	-	+	+	+	+	+	+	+	+	+	+	1	0.03%
+	+	+	+	+	+	+	-	+	-	+	-	-	+	-	+	+	1	0.03%
-	+	-	+	+	-	+	-	+	+	-	+	+	+	-	+	+	1	0.03%
+	+	+	+	+	-	+	+	+	-	+	-	-	+	-	+	+	1	0.03%
+	-	+	+	+	+	+	+	+	-	-	+	+	+	-	+	+	1	0.03%
-	+	-	+	-	+	+	+	+	+	+	+	+	+	+	+	+	1	0.03%
-	+	-	+	-	+	-	+	+	-	+	-	-	+	+	+	+	1	0.03%
-	+	-	+	+	+	+	-	+	-	+	+	+	+	-	+	+	1	0.03%
+	+	+	+	+	+	+	-	+	-	-	+	-	+	-	+	+	1	0.03%
+	+	-	+	+	-	+	-	+	+	-	+	+	+	-	+	+	1	0.03%
+	+	-	+	-	+	+	+	+	-	+	-	-	+	+	+	+	1	0.03%
-	+	-	+	-	+	+	+	+	+	+	+	+	-	-	+	+	1	0.03%
+	+	-	+	+	+	+	+	+	-	-	+	+	+	+	+	+	1	0.03%
+	+	-	+	+	-	+	-	+	-	-	+	+	+	+	-	+	1	0.03%
+	-	+	+	+	-	+	-	+	-	-	+	+	+	+	+	+	1	0.03%
+	+	+	+	+	+	+	-	+	-	-	+	-	+	+	+	+	1	0.03%
+	-	+	+	+	+	+	+	+	-	-	+	+	-	-	+	+	1	0.03%
+	+	-	+	+	-	+	-	+	-	-	+	-	+	+	-	+	1	0.03%
+	-	+	+	+	+	+	+	+	-	+	-	-	+	+	+	+	1	0.03%
+	-	+	+	+	-	+	-	+	-	+	+	-	+	+	-	+	1	0.03%
-	+	-	+	+	-	+	-	-	+	-	+	-	+	-	+	+	1	0.03%
+	+	-	+	+	-	+	-	+	+	+	+	-	+	+	-	+	1	0.03%
-	+	-	+	-	+	-	+	+	-	-	+	-	+	-	+	-	1	0.03%
+	+	+	-	+	+	+	+	+	-	+	+	-	+	-	+	+	1	0.03%
+	-	+	+	+	+	+	+	+	+	+	-	-	+	-	+	+	1	0.03%
+	+	-	+	-	+	-	+	+	+	+	-	-	+	+	+	+	1	0.03%
-	+	-	+	+	-	+	+	+	-	+	-	-	+	-	+	+	1	0.03%
-	+	-	+	+	-	+	+	+	-	-	+	-	+	-	+	+	1	0.03%
-	+	-	+	+	+	-	+	+	-	+	+	-	+	+	+	+	1	0.03%
+	+	-	+	-	+	-	+	+	-	-	+	+	+	-	+	+	1	0.03%
+	+	-	+	+	-	+	-	+	-	+	-	+	+	+	-	+	1	0.03%
+	+	-	+	+	+	+	+	+	-	+	-	+	-	-	+	+	1	0.03%
+	+	-	+	+	+	+	+	+	-	-	+	-	+	+	-	+	1	0.03%
-	+	-	+	+	-	+	-	-	+	+	-	+	+	+	+	+	1	0.03%
+	-	+	-	+	+	+	-	+	-	+	+	-	+	-	+	+	1	0.03%
+	+	+	+	-	+	+	+	+	-	+	-	-	+	-	+	+	1	0.03%
+	-	-	+	+	+	+	-	+	+	+	+	-	+	-	+	+	1	0.03%
+	-	-	+	+	+	+	-	+	+	+	+	-	+	+	+	+	1	0.03%
+	-	-	+	-	+	+	+	+	+	-	+	-	+	-	+	+	1	0.03%
+	+	+	+	+	+	+	-	+	+	+	-	-	+	-	+	+	1	0.03%
+	+	-	+	+	+	-	+	+	-	+	-	-	+	-	+	+	1	0.03%
-	+	+	+	+	+	+	+	+	-	+	-	-	+	+	+	+	1	0.03%
+	-	-	+	+	+	+	+	-	+	+	-	-	+	-	+	+	1	0.03%
+	-	-	+	-	+	-	+	+	+	-	+	-	+	-	+	+	1	0.03%

+	+	-	+	+	+	+	+	+	+	-	+	+	+	+	+	+	1	0.03%
+	+	-	+	-	+	+	+	+	-	-	+	+	+	+	+	+	1	0.03%
+	+	+	+	+	+	+	-	+	+	+	+	-	+	+	+	+	1	0.03%
+	-	+	-	+	+	+	+	+	-	+	+	-	+	-	+	+	1	0.03%
+	-	-	+	-	+	+	+	+	+	+	+	+	+	-	+	+	1	0.03%
-	+	-	+	-	+	-	+	+	+	+	-	-	+	-	+	+	1	0.03%
-	+	+	+	+	-	+	-	+	+	-	+	-	+	-	+	+	1	0.03%
+	-	-	+	+	+	+	+	+	-	-	+	-	+	+	-	+	1	0.03%
+	-	-	+	+	-	+	+	+	-	+	+	-	+	-	+	+	1	0.03%
+	+	-	+	-	+	-	+	+	-	+	-	+	+	-	+	+	1	0.03%
+	-	-	+	-	+	-	+	+	-	+	-	+	+	+	+	+	1	0.03%
+	-	-	+	+	+	+	+	+	-	+	-	+	+	+	+	+	1	0.03%
-	+	-	+	+	-	+	-	+	-	+	+	+	+	-	+	-	1	0.03%
+	-	+	-	+	-	+	-	+	+	+	-	-	+	-	+	+	1	0.03%
+	+	-	+	+	-	+	-	+	-	+	+	-	+	-	+	-	1	0.03%
+	+	-	+	-	+	-	+	+	+	+	+	+	+	-	+	+	1	0.03%
+	+	-	+	+	-	+	-	-	+	+	+	-	+	-	+	+	1	0.03%
+	+	+	+	+	+	+	-	+	-	+	+	-	+	+	+	+	1	0.03%
+	-	+	+	+	-	+	-	+	+	-	+	-	+	-	+	+	1	0.03%
+	-	-	+	+	+	+	-	+	-	+	-	-	+	+	+	+	1	0.03%
-	+	-	+	+	+	+	+	+	-	-	+	+	-	-	+	+	1	0.03%
-	+	-	+	-	+	-	+	+	-	+	-	+	+	-	+	+	1	0.03%
+	-	+	+	+	-	+	-	+	+	+	+	-	+	+	+	+	1	0.03%
+	+	-	+	-	+	+	-	+	-	-	+	-	+	-	+	+	1	0.03%
-	+	-	+	+	+	+	+	+	+	+	-	+	+	-	+	+	1	0.03%
+	-	+	-	+	-	+	-	+	-	+	+	-	+	-	+	+	1	0.03%
+	-	-	+	+	+	+	+	+	+	+	-	+	+	-	+	+	1	0.03%
-	+	-	+	+	+	+	-	+	+	+	+	-	+	+	-	+	1	0.03%
-	+	-	+	+	+	-	+	+	-	-	+	-	+	-	+	+	1	0.03%
-	+	-	+	+	-	+	-	+	-	-	+	+	+	+	+	+	1	0.03%
+	+	+	-	+	-	+	-	+	-	-	+	-	+	-	+	+	1	0.03%
-	+	-	+	+	-	+	-	+	-	+	-	-	+	+	-	+	1	0.03%
+	-	+	+	-	+	+	+	+	-	+	-	-	+	-	+	+	1	0.03%
+	-	+	+	+	+	+	-	+	-	+	-	-	+	-	+	+	1	0.03%
+	-	-	+	+	-	+	-	+	-	+	+	+	-	+	+	+	1	0.03%
-	+	-	+	-	+	-	+	-	+	+	+	+	+	+	+	+	1	0.03%
+	-	-	+	-	+	+	-	+	-	-	+	-	+	-	+	+	1	0.03%
+	+	-	+	+	-	+	-	+	-	+	-	+	+	+	+	+	1	0.03%
+	+	+	+	-	+	-	+	+	+	+	-	-	+	-	+	+	1	0.03%
+	-	-	+	+	+	+	+	+	-	+	+	-	+	+	-	+	1	0.03%
+	+	-	+	+	+	+	-	+	+	+	-	-	+	+	+	+	1	0.03%
-	+	-	+	+	+	+	+	+	-	-	+	-	+	+	-	+	1	0.03%
+	+	+	+	+	+	+	+	+	-	+	+	+	+	-	+	+	1	0.03%
+	+	+	+	-	+	-	+	+	-	+	-	-	+	-	+	+	1	0.03%
+	-	+	+	+	+	+	+	+	-	+	+	+	+	+	+	+	1	0.03%
+	-	-	+	+	+	+	+	+	+	-	+	+	+	-	+	+	1	0.03%
-	+	-	+	+	+	+	+	+	-	+	-	+	+	+	+	+	1	0.03%
+	+	-	+	+	+	+	+	+	+	+	+	-	+	+	-	+	1	0.03%

+	+	+	+	+	+	+	-	+	-	+	+	-	+	-	+	+	1	0.03%
+	+	+	+	+	-	+	-	+	-	+	+	+	-	-	+	+	1	0.03%
+	-	-	+	+	-	+	-	+	-	+	+	-	+	+	-	+	1	0.03%
+	+	+	+	-	+	-	+	+	-	+	+	+	+	-	+	+	1	0.03%
+	+	-	+	+	-	+	-	+	-	+	+	+	-	-	+	+	1	0.03%
+	+	-	+	+	+	+	-	+	-	-	+	-	+	+	-	+	1	0.03%
+	+	-	+	-	+	+	-	+	+	-	+	-	+	-	+	+	1	0.03%
+	-	+	+	-	+	+	+	+	+	-	+	+	+	-	+	+	1	0.03%
+	+	-	+	+	-	+	-	-	+	+	-	+	+	-	+	+	1	0.03%
-	+	-	+	+	-	+	-	+	-	+	-	+	+	+	+	+	1	0.03%
-	+	-	+	+	-	+	-	+	-	+	-	+	+	+	-	+	1	0.03%
+	+	-	+	+	-	+	+	+	+	+	+	-	+	-	+	+	1	0.03%
+	-	-	+	+	+	+	+	+	-	-	+	+	+	+	+	+	1	0.03%
+	+	-	+	-	+	-	+	+	-	-	+	+	-	-	+	+	1	0.03%
+	+	-	+	+	-	+	-	+	-	-	+	+	-	-	+	+	1	0.03%
+	+	-	+	+	-	+	+	+	-	-	+	-	+	-	+	+	1	0.03%
-	+	-	+	+	+	+	-	+	-	-	+	-	+	+	+	+	1	0.03%
+	+	-	+	+	+	+	+	+	-	-	+	+	+	-	+	-	1	0.03%
+	+	+	+	+	+	+	+	+	-	-	+	-	+	+	-	+	1	0.03%
-	+	-	+	+	+	+	+	-	+	-	+	-	+	-	+	+	1	0.03%
+	-	-	+	-	+	-	+	+	+	+	-	-	+	-	+	+	1	0.03%
+	-	+	-	+	-	+	-	+	+	+	+	-	+	+	+	+	1	0.03%
-	+	+	+	-	+	-	+	+	+	+	-	-	+	-	+	+	1	0.03%
-	+	-	+	-	+	-	+	+	+	-	+	-	+	+	+	+	1	0.03%
+	-	-	+	-	+	+	+	+	-	+	-	-	+	-	+	+	1	0.03%
+	-	-	+	+	+	+	-	+	-	+	+	+	+	-	+	+	1	0.03%
-	+	+	+	+	-	+	-	+	-	+	-	-	+	+	+	+	1	0.03%
-	+	-	+	+	+	+	-	+	+	+	-	-	+	+	+	+	1	0.03%
+	+	-	+	+	-	+	+	+	-	+	-	-	+	-	+	+	1	0.03%
+	-	-	+	+	+	-	+	+	+	+	-	-	+	-	+	+	1	0.03%
+	+	+	+	+	-	+	-	+	-	+	+	-	+	-	+	+	1	0.03%
-	+	+	+	+	+	+	+	+	-	-	+	+	+	-	+	+	1	0.03%
-	+	-	+	-	+	+	+	+	-	+	-	+	+	-	+	+	1	0.03%
+	+	+	+	+	+	+	-	+	-	-	+	+	+	-	+	+	1	0.03%
+	+	+	-	+	+	+	+	+	-	-	+	-	+	-	+	+	1	0.03%
-	+	+	+	+	+	+	-	+	-	+	-	+	+	-	+	+	1	0.03%
-	+	-	+	-	+	+	-	+	-	+	-	-	+	-	+	+	1	0.03%
-	+	+	+	+	+	+	+	+	-	+	-	+	+	-	+	+	1	0.03%
+	+	+	+	+	+	-	+	+	-	-	+	-	+	-	+	+	1	0.03%
-	+	+	+	+	+	+	+	+	-	+	+	+	+	-	+	+	1	0.03%
-	+	-	+	+	+	+	-	+	-	-	+	+	+	+	-	+	1	0.03%
-	+	-	+	-	+	-	+	+	-	+	+	-	+	-	+	-	1	0.03%
+	+	-	+	-	+	-	+	+	+	-	+	+	+	-	+	+	1	0.03%
-	+	+	+	+	+	+	+	+	+	+	-	-	+	-	+	+	1	0.03%
-	+	-	+	+	-	+	+	+	+	-	+	+	+	-	+	+	1	0.03%
+	+	-	+	+	+	+	-	+	+	-	+	+	+	+	+	+	1	0.03%
+	+	-	+	+	+	+	+	+	+	+	+	-	+	+	+	+	1	0.03%
-	+	+	-	+	+	+	+	+	+	+	+	-	+	-	+	+	1	0.03%

-	+	-	+	+	-	+	-	+	+	-	+	-	+	+	+	+	1	0.03%
-	+	+	+	-	+	+	+	+	+	-	+	-	+	-	+	+	1	0.03%
-	+	-	+	+	-	+	-	-	+	+	+	-	+	+	+	+	1	0.03%
-	+	-	+	+	-	+	-	-	+	+	-	-	+	-	+	+	1	0.03%

Supplementary Table S2. Frequencies of alleles associated with rare blood group phenotypes.

Group	Phenotype	Nucleotide	Amino acid	Korean*				East Asian**				European**			
				Numbers	Total allele count	Frequency	Predicted phenotype frequency	Numbers	Total allele count	Frequency	Predicted phenotype frequency	Numbers	Total allele count	Frequency	Predicted phenotype frequency
P1PK	p	c.1029dup	p.Thr344Hisfs103Ter	1	9612	0.010%									
		c.301del	p.Ala101Profs13Ter	1	9612	0.010%		1	14570	0.007%					
		c.752C>T	p.Pro251Leu									7	137586	0.005%	
		c.560G>A	p.Gly187Asp				0.000004%				0.000000%	1	134754	0.001%	0.000004%
		c.548T>A	p.Met183Lys									11	134788	0.008%	
		c.498G>A	p.Trp166Ter									1	134524	0.001%	
		c.299C>T	p.Ser100Leu									2	135292	0.001%	
		c.241_243del	p.Phe81del									7	153326	0.005%	
Kell	Null	c.715G>T	p.Glu239Ter	13	11724	0.111%		6	16136	0.037%					
		c.712C>T	p.Gln238Ter	2	11722	0.017%									
		c.299G>C	p.Cys100Ser	2	9612	0.021%									
		c.233+1G>A	-	1	9612	0.010%		1	16130	0.006%		19	154020	0.012%	
		c.184dup	p.Ser62Phefs17Ter	2	11730	0.017%		4	14574	0.027%					
		c.2098C>T	p.Arg700Ter									4	135120	0.003%	
		c.2023C>T	p.Arg675Ter									51	138622	0.037%	
		c.1726G>C	p.Gly576Arg									1	134880	0.001%	
		c.1708G>A	p.Val570Met									9	153708	0.006%	
		c.1546C>T	p.Arg516Ter					2	16132	0.012%		7	154150	0.005%	
		c.1477C>T	p.Gln493Ter									1	135402	0.001%	
		c.1474C>T	p.Arg492Ter									2	135392	0.001%	
		c.1420C>T	p.Gln474Ter				0.000310%				0.000176%	6	154210	0.004%	0.000142%
		c.1253T>C	p.Phe418Ser									1	154176	0.001%	
		c.1216C>T	p.Arg406Ter									6	134582	0.004%	
		c.1130T>C	p.Leu377Pro									1	135338	0.001%	
		c.924+1G>T	-									7	149812	0.005%	
		c.904del	p.Val302SerfsTer28									8	132878	0.006%	
		c.574C>T	p.Arg192Ter									4	135338	0.003%	
		c.436del	p.Glu146ArgfsTer43									1	18900	0.005%	
		c.382C>T	p.Arg128Ter									2	154278	0.001%	
		c.246T>A	p.Cys82Ter									1	135346	0.001%	
		c.223+1G>T	-									1	135132	0.001%	
		c.71G>A	p.Trp24Ter									1	135374	0.001%	
	K0 phenotype	c.1719C>T	p.Gly573Gly					8	16126	0.050%		33	153710	0.021%	

Kidd	Jk(a-b-)	c.342-1G>A	-	35	20182	0.173%		211	16132	1.308%		1	154206	0.001%	
		c.810G>A	p.Ala270Ala	6	20180	0.030%		3	16134	0.019%		11126	154144	7.218%	
		c.956C>T	p.Thr319Met	1	16426	0.006%						3	135342	0.002%	
		c.190C>T	p.Arg64Trp				0.000438%				0.017780%	1	135344	0.001%	0.521999%
		c.202C>T	p.Gln68Ter									2	135362	0.001%	
		c.582C>G	p.Tyr194Ter									3	154266	0.002%	
		c.811+5G>A	-					1	14550	0.007%					
Colton	Co(b+)	c.134C>T	p.Ala45Val	2	13250	0.015%	0.000002%				0.000000%	6377	153968	4.142%	0.171543%
Indian	In(a+b-)	c.137G>C	p.Arg46Pro	2	9612	0.021%	0.000004%				0.000000%	3	154212	0.002%	0.000000%
Ok	Ok(a-)	c.274G>A	p.Glu92Lys	92	20002	0.460%	0.002116%				0.000000%	2	151370	0.001%	0.000000%
RHAG	Rhnull	157+1G>A	-	1	9612	0.010%						8	154186	0.005%	
		c.946-2A>G	-				0.000001%				0.000000%	3	135050	0.002%	0.000001%
		c.836G>A	p.Gly279Glu									1	135358	0.001%	
		c.310C>T	p.Gln104Ter									1	135344	0.001%	
JR	Jr(a-)	c.376C>T	p.Gln126Ter	325	20066	1.620%		51	16114	0.316%					
		c.2T > C	-	25	20154	0.124%									
		c.1723C > T	p.Arg575Ter	2	20200	0.010%		17	16136	0.105%		3	154142	0.002%	
		c.706C>T	p.Arg236Ter	1	13358	0.007%		7	16132	0.043%		48	154116	0.031%	
		c.1111_1112del	p.Thr371LeufsTer20												
		c.986_987del	p.Ile329ArgfsTer19												
		c.875_878dup	p.Phe293LeufsTer8				0.031014%				0.002288%				0.000065%
		c.791_792del	p.Leu264HisfsTer14									11	154048	0.007%	
		c.784G>T	p.Gly262Ter									6	154032	0.004%	
		c.736C>T	p.Arg246Ter									20	154138	0.013%	
		c.439C>T	p.Arg147Trp									18	154062	0.012%	
		c.337C>T	p.Arg113Ter					1	14560	0.007%		13	134946	0.010%	
		c.263+1G>A	-					1	16128	0.006%		3	154146	0.002%	
LAN	Lan-	c.459del	p.Trp154GlyfsTer96Ter	15	20038	0.075%		18	16132	0.112%					
		c.1118_1124del	p.Ala373GlyfsTer47Ter	2	9612	0.021%		2	14058	0.014%		3	124194	0.002%	
		c.85_87del	p.Phe29del	2	13126	0.015%		1	15902	0.006%		6	150792	0.004%	
		c.1985_1986del	p.Leu662ProfsTer15									7	135354	0.005%	
		c.1942C>T	p.Arg648Ter									9	154300	0.006%	
		c.1533_1543dup	p.Leu515ProfsTer43				0.000123%				0.000174%	6	154236	0.004%	0.001151%
		c.1236G>A	p.Trp412Ter									1	135346	0.001%	
		c.953_956del	p.Gly318AlafsTer8									2	135262	0.001%	
		c.718C>T	p.Arg240Ter									3	133268	0.002%	
		c.717G>A	p.Trp239Ter									45	152216	0.030%	
		c.574C>T	p.Arg192Trp									436	153544	0.284%	

KANNO	KANNO1-	c.655G>A	p.Glu219Lys	1033	20160	5.124%	0.262555%	504	16130	3.125%	0.097632%	10	154220	0.006%	0.000000%
SID	Sd(a-)	c.1396T>C	p.Cys466Arg	856	20112	4.256%	0.181149%	428	16124	2.654%	0.071783%	17104	154078	11.101%	5.931266%
		c.1134+5G>A	-					3	16124	0.019%		18310	153658	11.916%	
		c.1307A>G	p.Gln436Arg					1	16128	0.006%		1	154040	0.001%	
		c.1567C>T	p.Arg523Trp									2059	154048	1.337%	

*Data analyzed from Korean Genome and Epidemiology Study, Genome Aggregation Database (gnomAD), and Korean Variant Archive.
**Data analyzed from gnomAD.

Supplementary Table S3. Comparison of predicted blood group antigen phenotype frequencies with previous Korean studies.

		KOGES WGS data (n = 2,897)		Shin et al.(2018) (n = 252)		Jekarl et al.(2019) (n=145)	
Group	Phenotype	Individuals	Frequency	Individuals	Frequency	Individuals	Frequency
MN	MM	717	24.75%	73	28.97%	33	22.76%
	MN	1310	45.22%	124	49.21%	77	53.1%
	NN	870	30.03%	55	21.83%	35	24.14%
S	ss	2627	90.68%	220	87.3%	131	90.34%
	Ss	259	8.94%	32	12.7%	14	9.66%
	SS	11	0.38%	0	0%	0	0%
C	cc	341	11.77%	35	13.89%	20	13.79%
	Cc	1305	45.05%	121	48.02%	63	43.45%
	CC	1251	43.18%	96	38.1%	62	42.76%
E	ee	1434	49.50%	121	48.02%	71	48.97%
	Ee	1199	41.39%	101	40.08%	62	42.76%
	EE	264	9.11%	30	11.9%	12	8.28%
Lutheran	Lu(a-b+)	2897	100%	248		145	100%
Kell	kk	2897	100%	252	100%	145	100%
Duffy	Fy(a+b-)	2468	85.19%	222	88.1%	131	90.34%
	Fy(a+b+)	417	14.39%	27	10.71%	13	8.97%
	Fy(a-b+)	12	0.41%	3	1.19%	1	0.69%
Kidd	Jk(a+b-)	650	22.44%	59	23.41%	38	26.21%
	Jk(a+b+)	1439	49.67%	124	49.21%	68	46.9%
	Jk(a-b+)	808	27.89%	69	27.38%	38	26.21%
Diego	Di(a+b-)	10	0.35%	1	0.4%	1	0.69%
	Di(a+b+)	315	10.87%	19	7.54%	13	8.97%
	Di(a-b+)	2572	88.78%	232	92.06%	131	90.34%
Yt	YT(a+b-)	2897	100%	252	100%	145	100%
Dombrock	Do(a+b-)	31	1.07%	4	1.59%	2	1.38%
	Do(a+b+)	551	19.02%	51	20.24%	21	14.48%
	Do(a-b+)	2315	79.91%	197	78.17%	122	84.14%
Colton	Co(a+b-)	2897	100%	252	100%	144	99.31%
	Co(a+b+)	0	0%	0	0%	1	0.69%
